# Potential Role of Lysine Acetylation and Autophagy in Brown Film Formation and Postripening of Lentinula edodes Mycelium

**DOI:** 10.1128/spectrum.02823-22

**Published:** 2023-06-22

**Authors:** Ting Chu, Junjun Shang, Huahua Jian, Chunyan Song, Ruiheng Yang, Dapeng Bao, Qi Tan, Lihua Tang

**Affiliations:** a National Engineering Research Centre of Edible Fungi, Key Laboratory of Edible Fungi Resources and Utilization (South), Ministry of Agriculture, Institute of Edible Fungi, Shanghai Academy of Agricultural Sciences, Shanghai, China; b School of Food Sciences and Technology, Shanghai Ocean University, Shanghai, China; c State Key Laboratory of Microbial Metabolism, School of Life Sciences and Biotechnology, Shanghai Jiao Tong University, Shanghai, China; Universidade de Brasilia

**Keywords:** *Lentinula edodes*, brown film formation, lysine acetylation, TCA, inhibitor, autophagy

## Abstract

Lentinula edodes is one of the most widely cultivated edible mushrooms in the world. When cultivated in sawdust, the surface mycelium of *L. edodes* needs a long postripening stage wherein it forms a brown film (BF) by secreting and accumulating pigments. BF formation is critical for the high quality and yield of fruiting bodies. Protein lysine acetylation (KAC) is an important post-translational modification that regulates growth and development. Previous studies have shown that deacetylase levels are significantly increased during BF formation in the postripening stage of *L. edodes*. The aim of this study was to assess the role of protein acetylation during BF formation. To this end, we compared the acetylome of *L. edodes* mycelia before and after BF formation using anti-acetyl antibody-based label-free quantitative proteomics. We identified 5,613 acetylation sites in 1,991 proteins, and quantitative information was available for 4,848 of these sites in 1,815 proteins. Comparative acetylome analysis showed that the modification of 699 sites increased and that of 562 sites decreased during BF formation. Bioinformatics analysis of the differentially acetylated proteins showed significant enrichment in the tricarboxylic acid (TCA) cycle and proteasome pathways. Furthermore, functional assays showed that BF formation is associated with significant changes in the activities of proteasome, citrate synthase, and isocitrate dehydrogenase. Consistent with this hypothesis, the lysine deacetylase inhibitor trichostatin (TSA) delayed autophagy and BF formation in *L. edodes*. Taken together, KAC and autophagy play important roles in the mycelial BF formation and postripening stage of *L. edodes*.

**IMPORTANCE** Mycelial BF formation and postripening of *L. edodes* affects the quality and quantity of its edible fruiting bodies. In this study, we explored the role of protein KAC in this biological process, with the aim of optimizing the cultivation and yield of *L. edodes*.

## INTRODUCTION

Lentinula edodes is an important edible fungus that is widely cultivated and consumed worldwide. It has a unique flavor, along with high nutritional and medicinal value. Polysaccharides isolated from *L. edodes* have anti-tumor, immunomodulatory, antioxidant, anti-inflammatory, antibacterial, and metabolic effects (1, 2). The cultivation of *L. edodes* includes four stages: growth of vegetative mycelia, formation of mycelial brown film (BF) and postripening, primordium initiation, and development of fruiting bodies. Mycelium BF formation and postripening have a significant impact on the yield, quality, and production cycle of *L. edodes*. In addition, the BF not only maintains the moisture level in the cultivation bag, but also inhibits bacterial and fungal contamination. In fact, inadequate BF formation in *L. edodes* mycelia increases the risk of infection by fungi such as *Trichoderma* (3). Through untargeted metabolite profiling, Tang et al. detected abundant anti-bacterial metabolites in the mycelial BF (4).

BF formation in *L. edodes* is affected by various environmental factors, and the mycelium normally produces BF when exposed to light (3, 5). Transcriptomic analysis has shown the involvement of genes related to photoreceptors, signal transduction pathways and melanogenesis in light-induced BF formation (5). Tang et al. found that the proteins differentially expressed during light-induced BF formation were involved in small-molecule metabolism and oxidative stress, among other physiological processes (6). Furthermore, Yoo et al. compared the transcriptomes of white mycelia, normal BFs and defective dark yellowish-brown films formed under different light conditions, and identified candidate genes closely associated with photoreceptor, G protein-coupled receptor signal transduction, tyrosinase-activated melanin synthesis, and cell wall degradation by glucanase, chitinase and laccase (3). Huang et al. confirmed that blue light promotes BF formation in *L. edodes* and increases its polysaccharide content (7). Furthermore, phosphoproteomics analysis of BFs formed under blue light identified several differentially phosphorylated proteins associated with light signal transduction, cell wall degradation and melanin synthesis, indicating a role of post-translational modifications (PTMs) in blue light-induced BF formation (8).

PTMs affect protein function by regulating their activity, cellular localization, and interactions with other proteins (9). Lysine acetylation (KAC) is a common PTM that plays an important role in cell morphogenesis, cell cycle regulation, and metabolic pathways (10–12). In a recent study, advanced mass spectrometry (MS)-based proteomics was applied to assess the role of protein acetylation in the growth and development of the pathogenic fungus Botrytis cinerea. The results showed that six proteins associated with virulence were acetylated, indicating that KAC regulates fungal pathogenesis (13). Analysis of the lysine acetylome of rice blast fungus (Magnaporthe oryzae) also showed that KAC plays an important role in its development and pathogenicity (14). Zhou and Wu (15) compared the acetylome of wild-type and GCN5-deficient strains of Fusarium graminearum, and identified a regulatory role of KAC in deoxynivalenol synthesis. Global acetylome analysis of Aspergillus flavus also revealed extensive involvement of acetylated proteins in glycolysis, gluconeogenesis, pentose phosphate pathway, citric acid cycle, and aflatoxin biosynthesis. Targeted mutagenesis further showed that KAC of the O-methyltransferase AflO might impair development, aflatoxin production, and pathogenicity (16). However, little is known regarding the role of protein acetylation in macrofungi, particularly edible fungi.

In a previous proteomics study, we found that the deacetylase RPD3 was significantly upregulated during mycelial BF formation and postripening in *L. edodes* (17). In addition, a growing body of evidence showed that acetylation can regulate autophagy (18, 19). The aim of the present study is to investigate the roles of acetylation and autophagy during mycelial BF formation and postripening in *L. edodes*. We employed label-free quantitative proteomics, acetylation enrichment techniques and high-resolution liquid chromatography (LC)-MS-based quantitative proteomics to compare the acetylomes of *L. edodes* mycelia before and after BF formation. Furthermore, we treated the *L. edodes* mycelia with trichostatin (TSA), a lysine deacetylase inhibitor, to confirm the role of acetylation and autophagy in these processes. Our findings not only widen the scope of comparative acetylome studies but also provide novel insights into the role of KAC and autophagy in the development of edible fungi.

## RESULTS

### BF formation in *L. edodes* mycelium is associated with global changes in protein acetylation.

**FIG 1:**
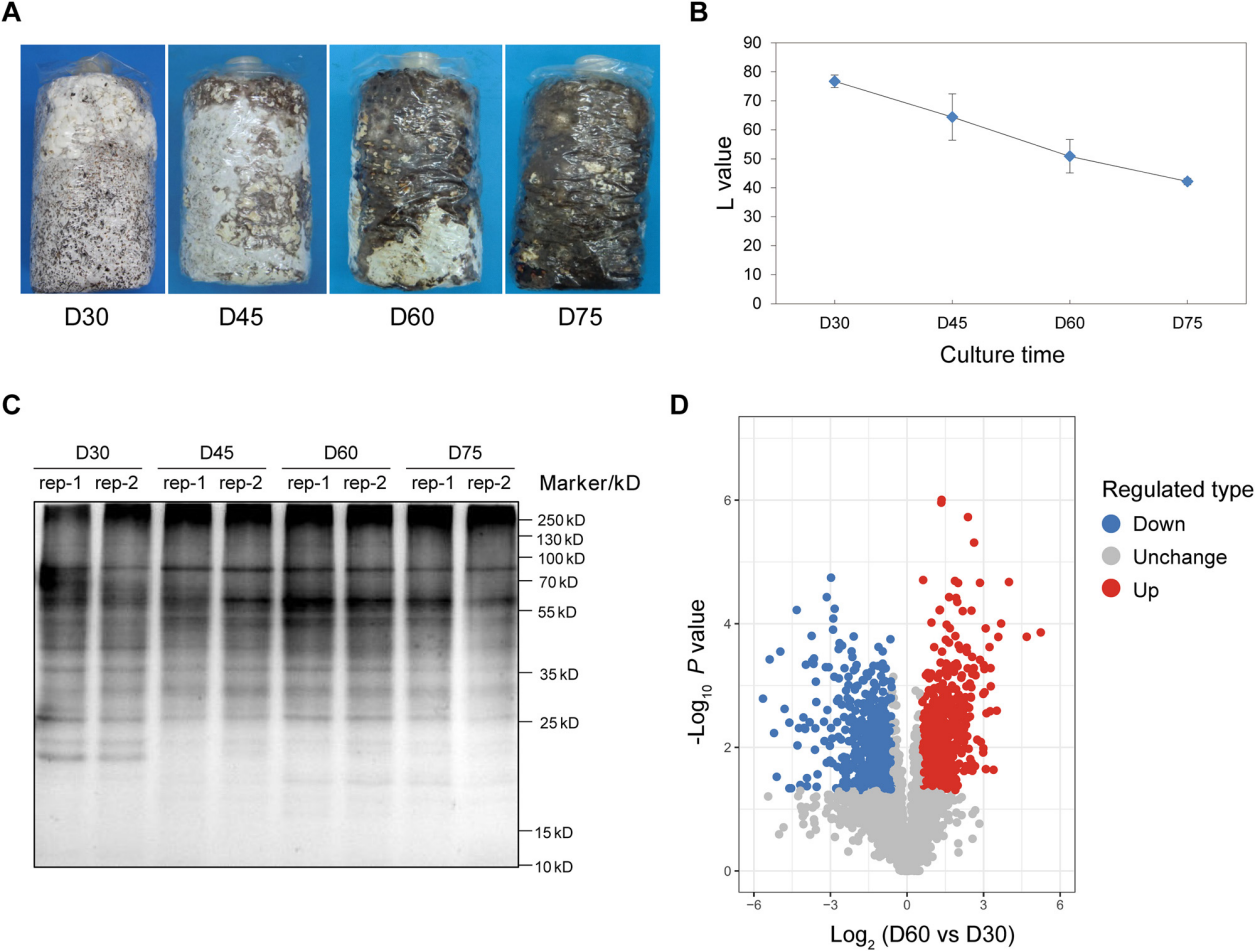
Overview of BF formation and acetylome analysis of *L. edodes* mycelium. (A) Representative images showing the morphology of the mycelium during BF formation and post-ripening. (B) The time-dependent change in the L value during the two stages. (C) Immunoblot showing levels of acetylated proteins during mycelium BF formation. (D) Volcano map of differentially acetylated sites (red points represent upregulated sites and blue points indicate downregulated sites). D30, day 30; D45, day 45; D60, day 60; D75, day 75.

To assess the physiological changes during *L. edodes* mycelial BF formation and postripening, we analyzed the morphological characteristics of the mycelium on days 30, 45, 60, and 75 of growth. As shown in Fig. 1A, the mycelium was white on day 30, while BF formation was widespread on day 60 and completed by day 75. The L value, an arbitrary unit of the white mycelium color, declined in a time-dependent manner. As shown in Fig. 1B, the average L value on day 30 was 76.63, which was indicative of very white mycelium, and decreased to 65.82, 51.86, and 42.14 on days 45, 60, and 75, respectively. And these L values have extremely significant difference among the four time points (Fig. S1). The change in protein KAC during BF formation was evaluated by measuring the pre-BF and post-BF levels of acetylated proteins using an anti-KAC antibody. As shown in Fig. 1C, there were multiple KAC protein bands at the different time points of mycelial growth, and there was a significant difference between day 30 and the other time points. The color of electrophoresis band of day 30 was darker, which indicated that the degree of acetylation was relatively high on day 30. Therefore, two samples, each of day 30 and day 60 mycelium, were used for analyzing protein acetylation. The workflow of the experimental procedures is shown in Fig. S2. We obtained 121,754 secondary fragment spectrograms, which were searched against the UniProt database to identify 39,691 usable effective spectra, corresponding to a spectral utilization rate of 32.6%. A total of 7,830 peptides and 5,546 acetylated peptides were identified through spectral analysis. In addition, we also identified 5,613 acetylation sites in 1,991 proteins, and quantitative information was available for 4,848 sites in 1,815 proteins. The precursor mass error of most spectra was within 10 ppm, which satisfies the criteria for high-precision MS. Most peptides consisted of 7 to 20 residues, which was consistent with trypsin activity (Fig. S3). These results indicated that our sample preparation met the quality standards. Overall, 754 proteins were differentially acetylated, which including 396 upregulated proteins and 358 downregulated proteins. And these differential acetylated proteins (DAPs) have 1,261 unique acetylated sites in the day 60 mycelium compared to day 30 mycelium (Fig. 1D; Fig. S4). The differentially acetylated proteins included many proteins, such as white collar-1, β-glucosidase, cytochrome P450, autophagy-related protein 8 (ATG8), and histones H3, H2B, H4, and H2A (Table S1). The number of KAC sites in these proteins varied from 1 to 25. A total of 831 (42%) proteins contained only one acetylation site, whereas 412 (31%), 265 (13%), and 483 (14%) proteins harbored two, three, and four or more acetylation sites, respectively (Fig. S5). The acetylated proteins were annotated based on Gene Ontology (GO) terms, protein domains, KEGG pathways, KOG category, and subcellular localization (Table S2).

### Motif and functional analyses of the DAPs associated with mycelial BF formation.

**FIG 2:**
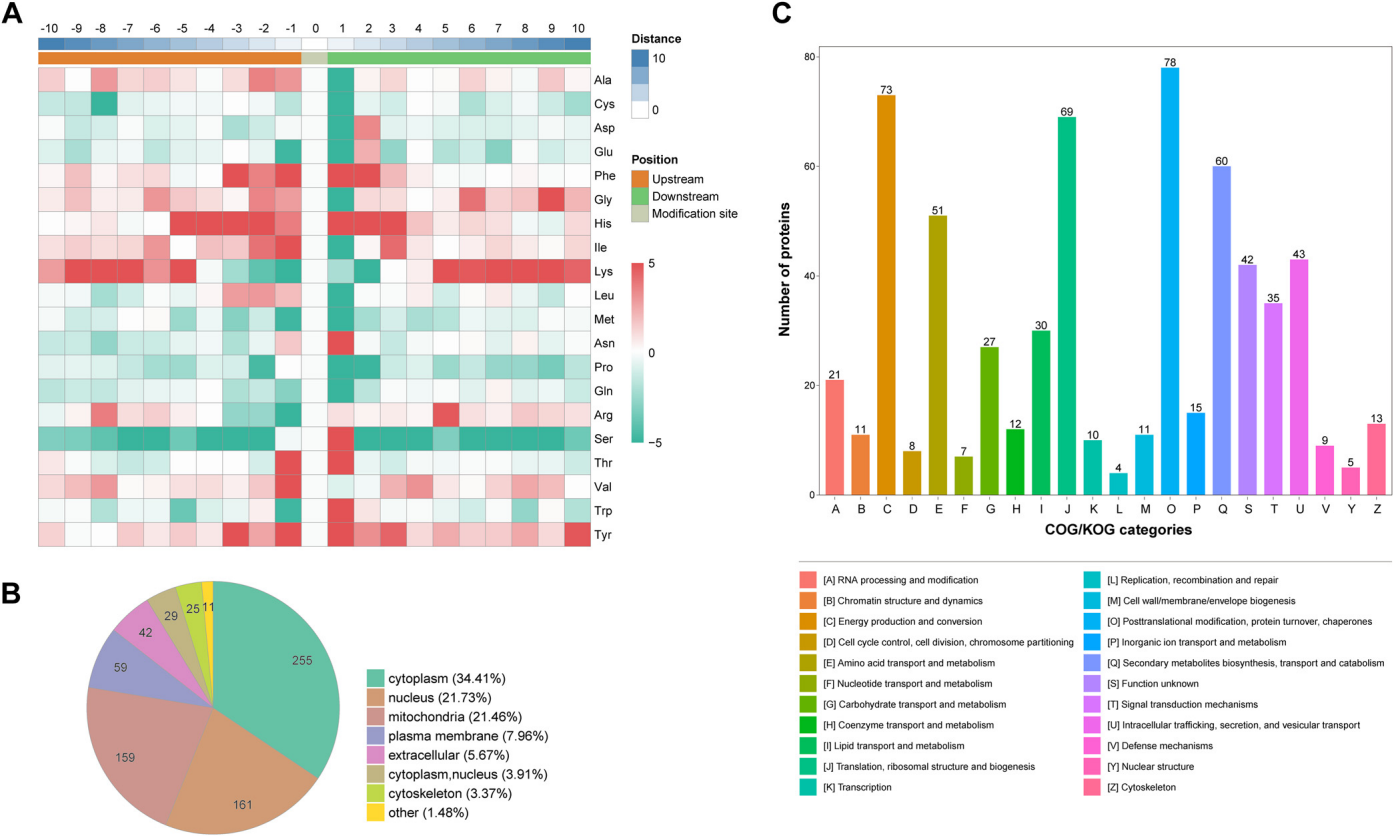
Motif analyses and functional characterization of acetylated proteins. (A) Heat map showing the frequency of different amino acids around the KAC sites. (B) Pie chart showing the subcellular distribution of DAPs. (C) COG/KOG functional classification statistics.

To determine the KAC pattern of the identified proteins, the amino acid sequences from −10 to +10 relative to the identified acetylation sites were analyzed using Motif-x software. As shown in the heatmap of amino acids around the KAC sites (Fig. 2A; Table S3), phenylalanine (F), histidine (H), isoleucine (I), lysine (K), asparagine (N), serine (S), threonine (T), valine (V), tryptophan (W), and tyrosine (Y) were highly enriched before and after the KAC site. These motifs might be conserved and necessary for KAC during the BF formation and postripening processes in *L. edodes* mycelia.

The subcellular localization of the DAPs was predicted using WoLF PSORT (Fig. 2B; Table S4). The DAPs were mainly distributed in the cytoplasm (34.41%), nucleus (21.73%), and mitochondria (21.46%), and the DAPs that were upregulated on day 60 consistently localized to the cytoplasm (33.33%), nucleus (25.25%), and mitochondria (18.69%). Furthermore, according to the clusters of orthologous groups (COG)/eukaryotic ortholog groups (KOG) functional classification (Fig. 2C; Table S5), the DAPs are mainly involved in the PTM of protein turnover chaperones (78), energy production and conversion (73), translation, ribosome structure and biogenesis (73), and secondary metabolite biosynthesis, transport and catabolism (73).

### Functional enrichment analysis of the DAPs.

**FIG 3:**
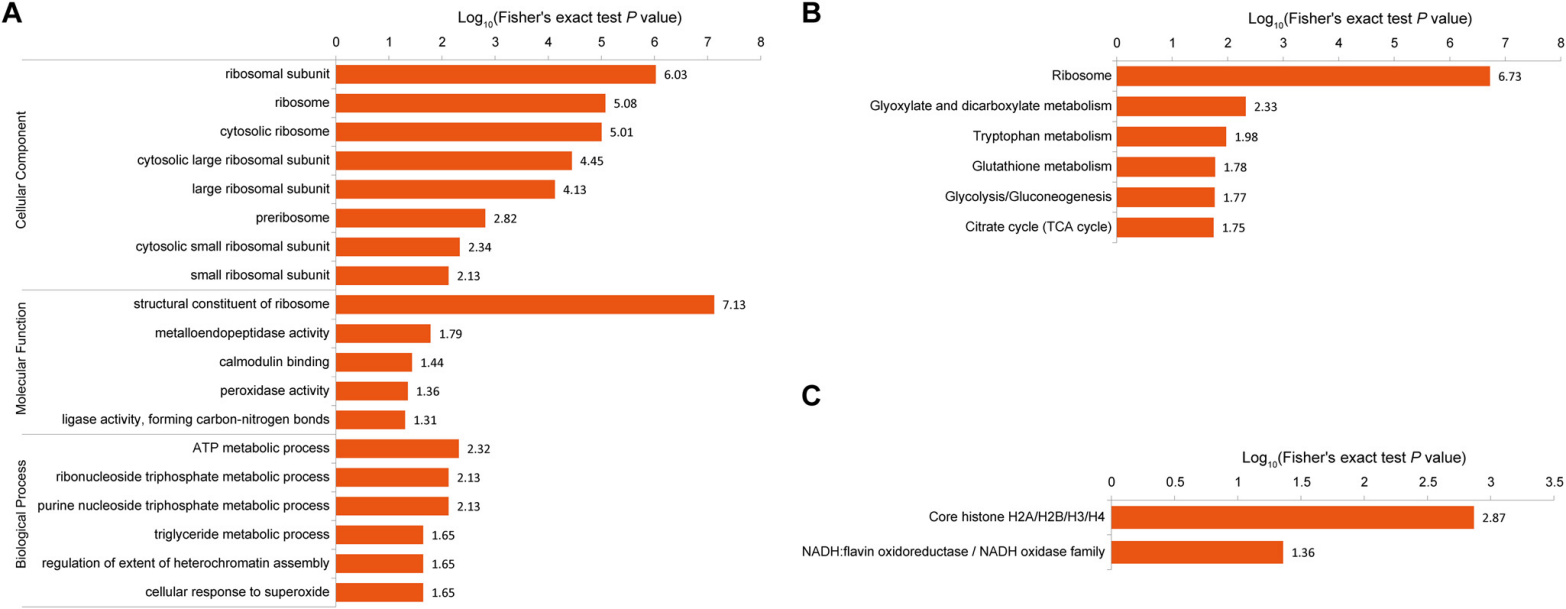
Functional enrichment analyses of the DAPs. (A) Significantly enriched GO terms related to biological processes, cellular components and molecular functions in the DAPs. (B) Results of KEGG pathway-based enrichment analysis. (C) Results of protein domain enrichment analysis.

GO enrichment analysis (Fig. 3A; Table S6) of the DAPs revealed significant enrichment of ATP metabolism, ribonucleoside triphosphate metabolism, purine nucleoside triphosphate metabolism (biological processes), ribosomes, cytoplasmic ribosomes, ribosomal subunits, large ribosomal subunits (cell components), and structural constituents of ribosomes (molecular functions).

KEGG analysis further showed that the downregulated DAPs were significantly enriched in the tricarboxylic acid (TCA) cycle (*P* = 0.000066), and the glyoxylate and dicarboxylate metabolism pathways (*P* = 0.000168), whereas the upregulated DAPs were significantly enriched in the ribosome (*P* = 3.537E-20), tryptophan metabolism (*P* = 0.0228), and glutathione metabolism pathways (*P* = 0.023098) (Fig. 3B; Table S7). These results indicate that the DAPs may play a role in mycelial metabolism during BF formation and postripening of *L. edodes*.

The protein domain enrichment analysis (Fig. 3C; Table S8) identified 52 functional domains. The core histone H2A/H2B/H3/H4 domains were significantly enriched in the DAPs, whereas the N α-terminal acetylation signal that marks proteins for proteasome-mediated degradation was significantly enriched in the downregulated DAPs. These result indicated that histone acetylation may play an epigenetic role in BF formation and postripening of *Lentinus edodes*.

### Proteasome proteins and TCA enzymes are acetylated during mycelial BF formation and postripening.

**FIG 4:**
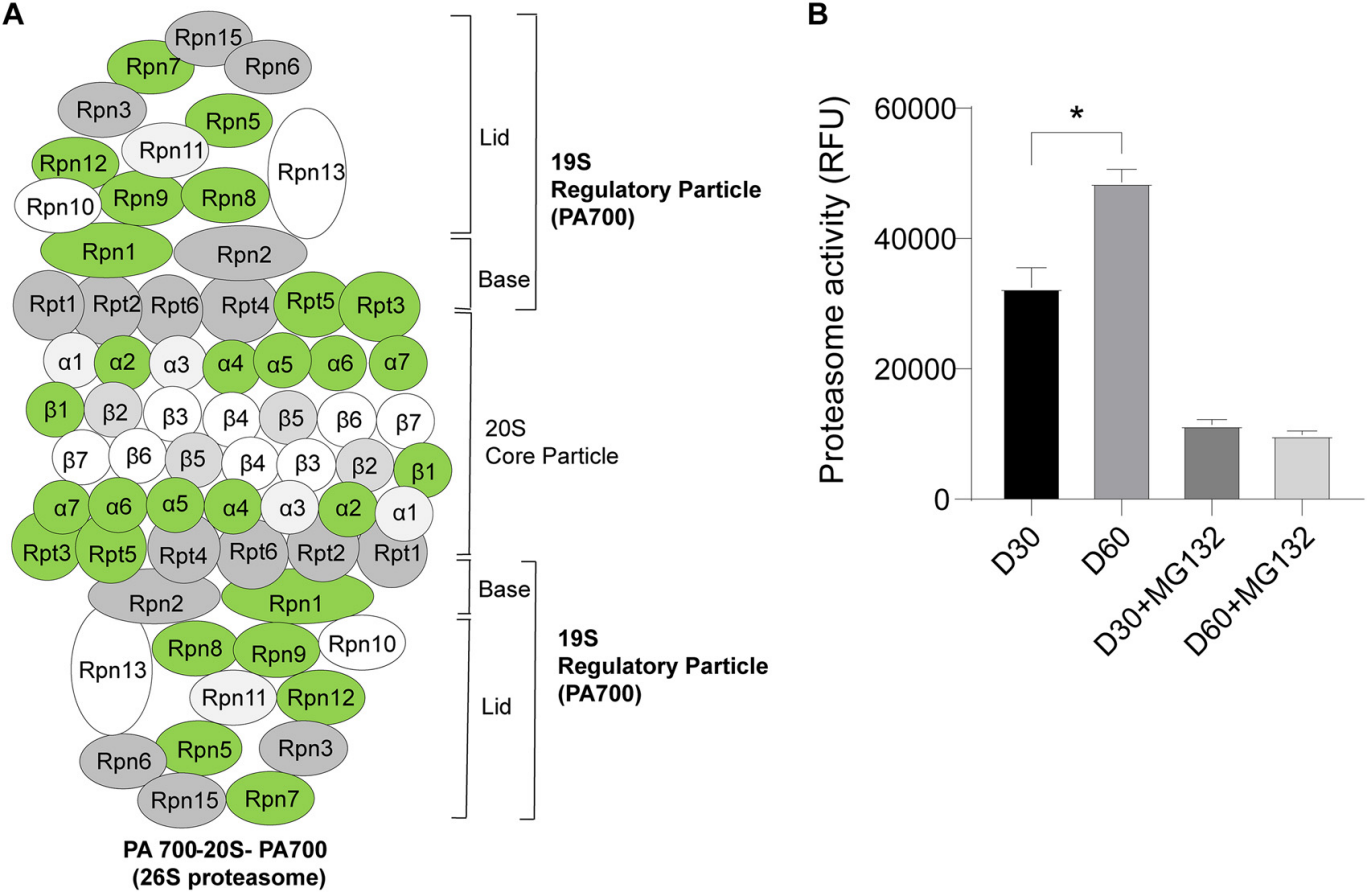
KEGG pathways significantly enriched in the differentially lysine-acetylated proteosomal proteins. (A) Diagram of the proteasome. (B) Analysis of 20S proteasome activity between day 30 and day 60. The green circle represents a significant downregulation of protein acetylation. RFU, relative fluorescence units. MG123 was used as an inhibitor of the 20S proteasome. D30, day 30; D60, day 60.

As shown in Fig. 4A, all KAC residues in the 20S catalytic core particle and the 19S regulatory particle of the proteasome were downregulated. Furthermore, proteasome activity was significantly higher on day 60 than on day 30 of cultivation (Fig. 4B), which suggested that the acetylation may regulate the activity of proteasomal proteins in mycelial BF formation and postripening of *L. edodes* (Table S9).

**FIG 5:**
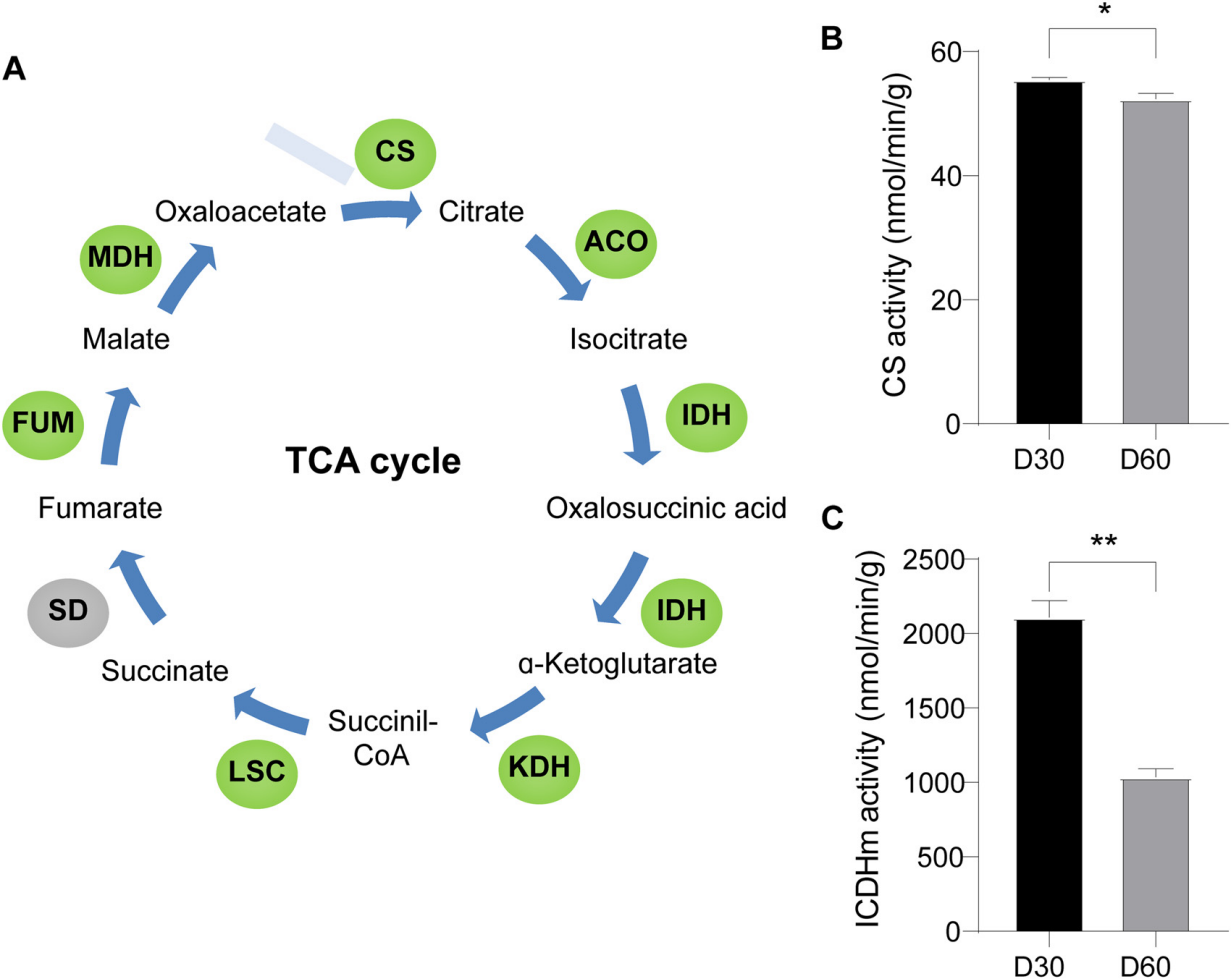
KEGG pathways significantly enriched in the differentially lysine-acetylated TCA cycle enzymes. (A) Diagram of the TCA cycle. (B) CS activity on day 30 and 60. (C) ICDHm activity on day 30 and 60. Green circle indicates that acetylation modification is significantly downregulated. CS, citrate synthase; ACO, aconitate hydratase; IDH, isocitrate dehydrogenase; KDH, α-oxoglutarate dehydrogenase; LSC, succinyl-CoA synthetase; SD, succinate dehydrogenase; FUM, fumarase; MDH, malate dehydrogenase. Green circle indicates significant downregulation of acetylation modification. D30, day 30; D60, day 60.

**FIG 6:**
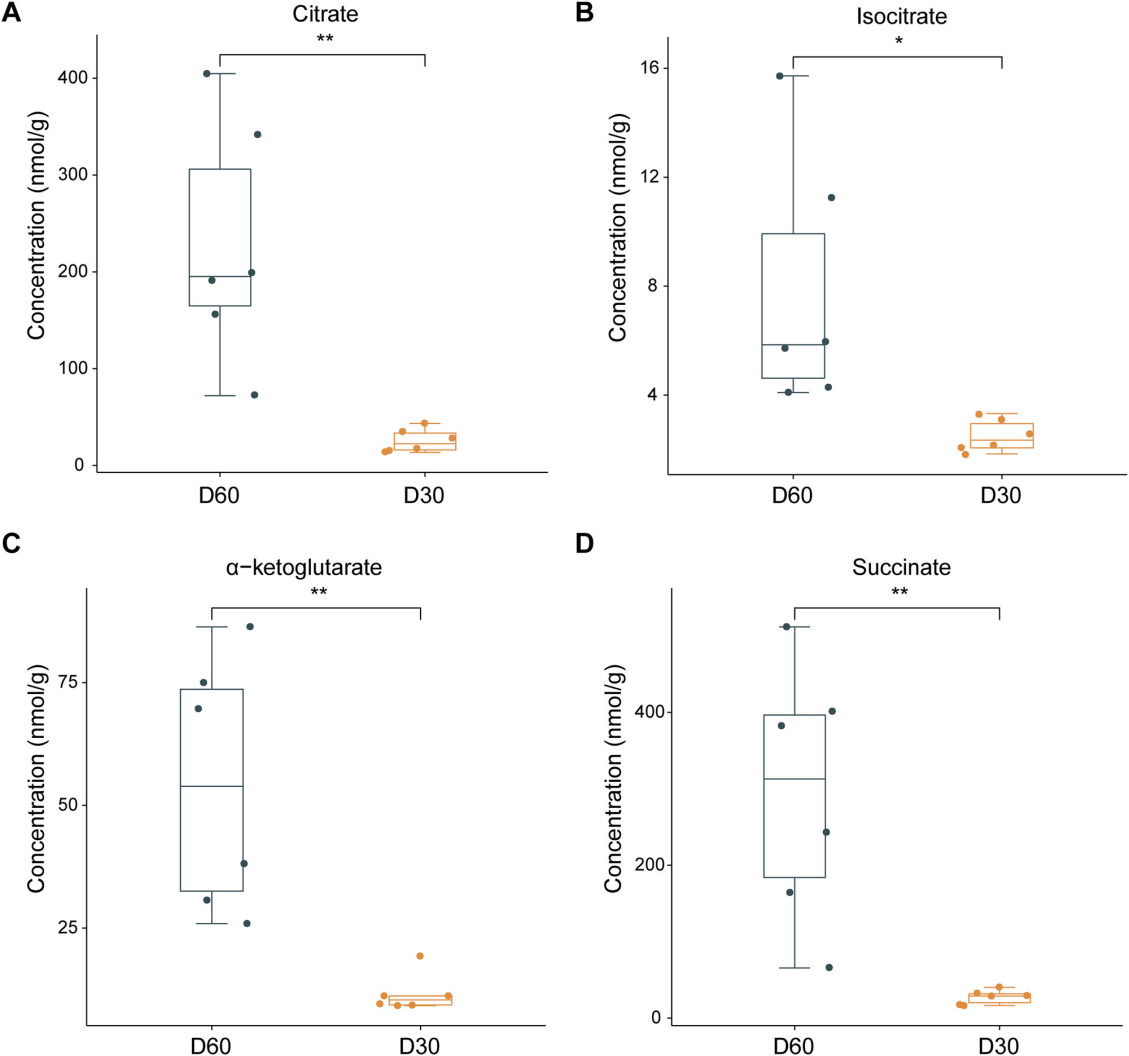
Content of intermediate metabolites of TCA cycle between day 30 and day 60 of mycelial growth. Bar graphs show the levels of (A) citrate, (B) isocitrate, (C) alpha-ketoglutarate, and (D) succinate. D30, day 30; D60, day 60.

Most of the acetylated enzymes in the TCA cycle, such as citrate synthase (CS) and isocitrate dehydrogenase (IDH), were also downregulated (Fig. 5A). As shown in Fig. 5B and C, the activities of CS and isocitrate dehydrogenase mitochondrial (ICDHm) were significantly decreased on day 60 (Table S9), which corresponded to an increase in the levels of the intermediate metabolites of TCA, including citrate, isocitrate, alpha-ketoglutarate and succinate (Fig. 6A to D; Table S10). These findings indicated that TCA cycle enzymes also are acetylated during mycelial BF formation and postripening in *L. edodes*.

### Autophagy-related proteins are acetylated during mycelial BF formation and postripening.

**FIG 7:**
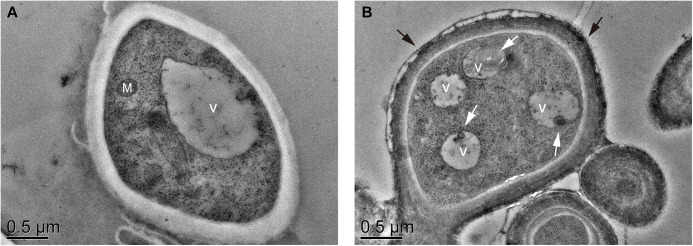
Representative TEM images of the ultrathin sections of (A) mycelia without BF on day 30 and (B) with BF on day 60. Black arrows: cell wall pigment layer; White arrows: autophagosome. M, mitochondrion; V, vacuole. Scale bars, 500 nm.

The ultrastructure of the mycelia before and after BF was examined by transmission electron microscopy (TEM). As shown in Fig. 7A, the mitochondria and vacuole were easily distinguishable prior to BF formation on day 30. The formation of BF (day 60) coincided with the appearance of distinct vacuoles, autophagosomes, and pigmented cell wall layer. The number of vacuoles increased and some autophagosomes were observed in mycelia with BF (day 60) (Fig. 7B). Thus, BF formation is associated with melanin deposition in the cell wall and generation of vacuoles, and the latter may be related to autophagy (17). The ubiquitin-like protein ATG8 forms an expanding structure with phosphatidyl ethanolamine (PE) to regulate autophagosome formation (20, 21). Acetylation of ATG8 was significantly upregulated on day 60 (3.109-fold higher than day 30 level, *P* = 0.0398), and the modification position was 13. Likewise, the acetylation of vacuolar protein sorting-associated protein 16 at position 497 was significantly upregulated on day 60 (1.773-fold higher than day 30 level, *P* = 0.0064) (Table S7). Taken together, the acetylation of autophagy-related proteins may play a role during BF formation.

### Inhibition of lysine deacetylases delayed BF formation in *L. edodes*.

**FIG 8:**
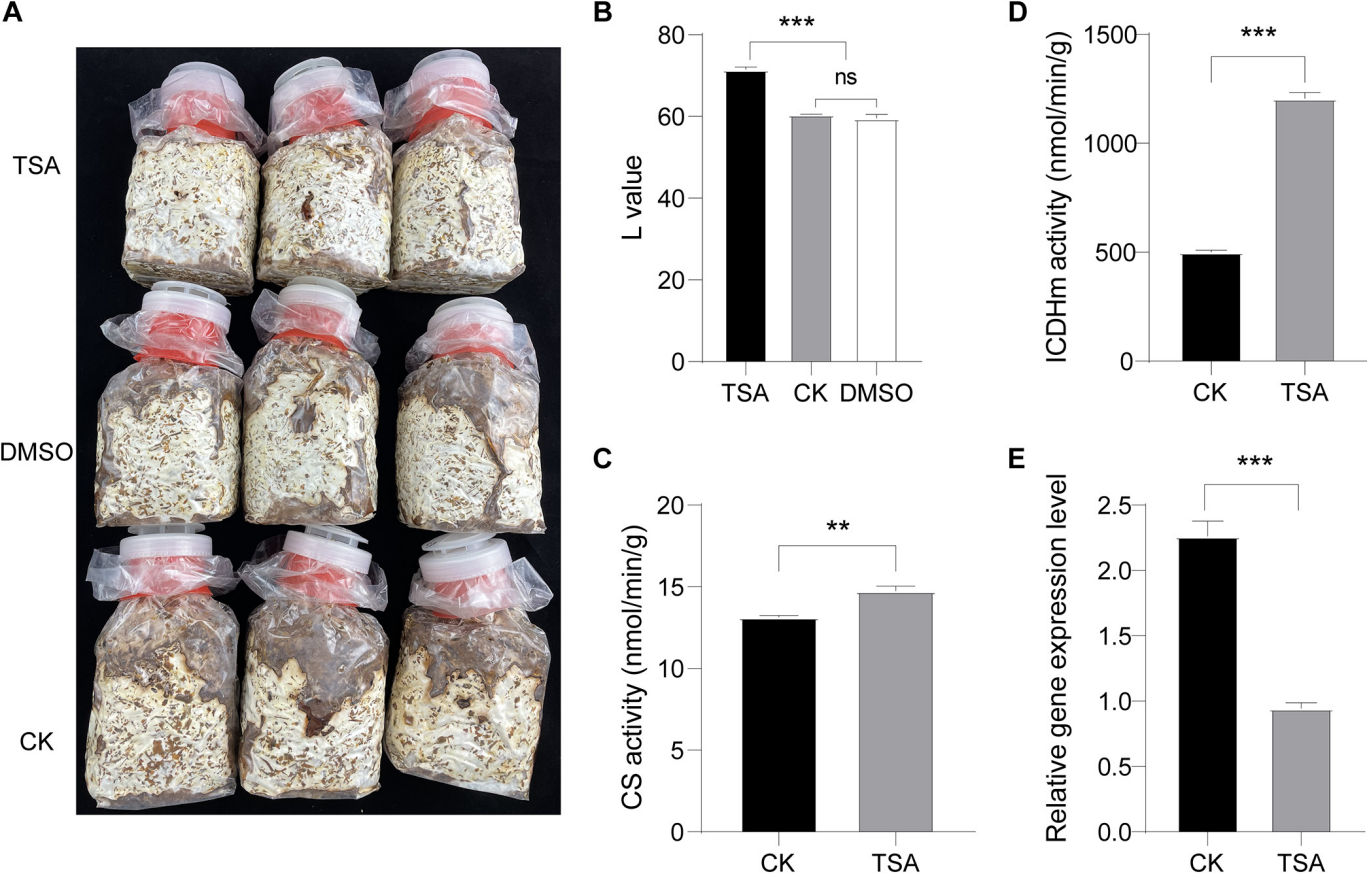
TSA delayed BF formation in *L. edodes*. (A) Representative images showing BF formation in TSA-treated, control (CK) and DMSO-treated groups. (B) The L values of the indicated groups. (C) CS activity in the TSA-treated and CK groups. (D) ICDHm activity in the TSA-treated and CK groups. (E) Relative *atg8* mRNA levels in the TSA-treated and CK groups.

**FIG 9:**
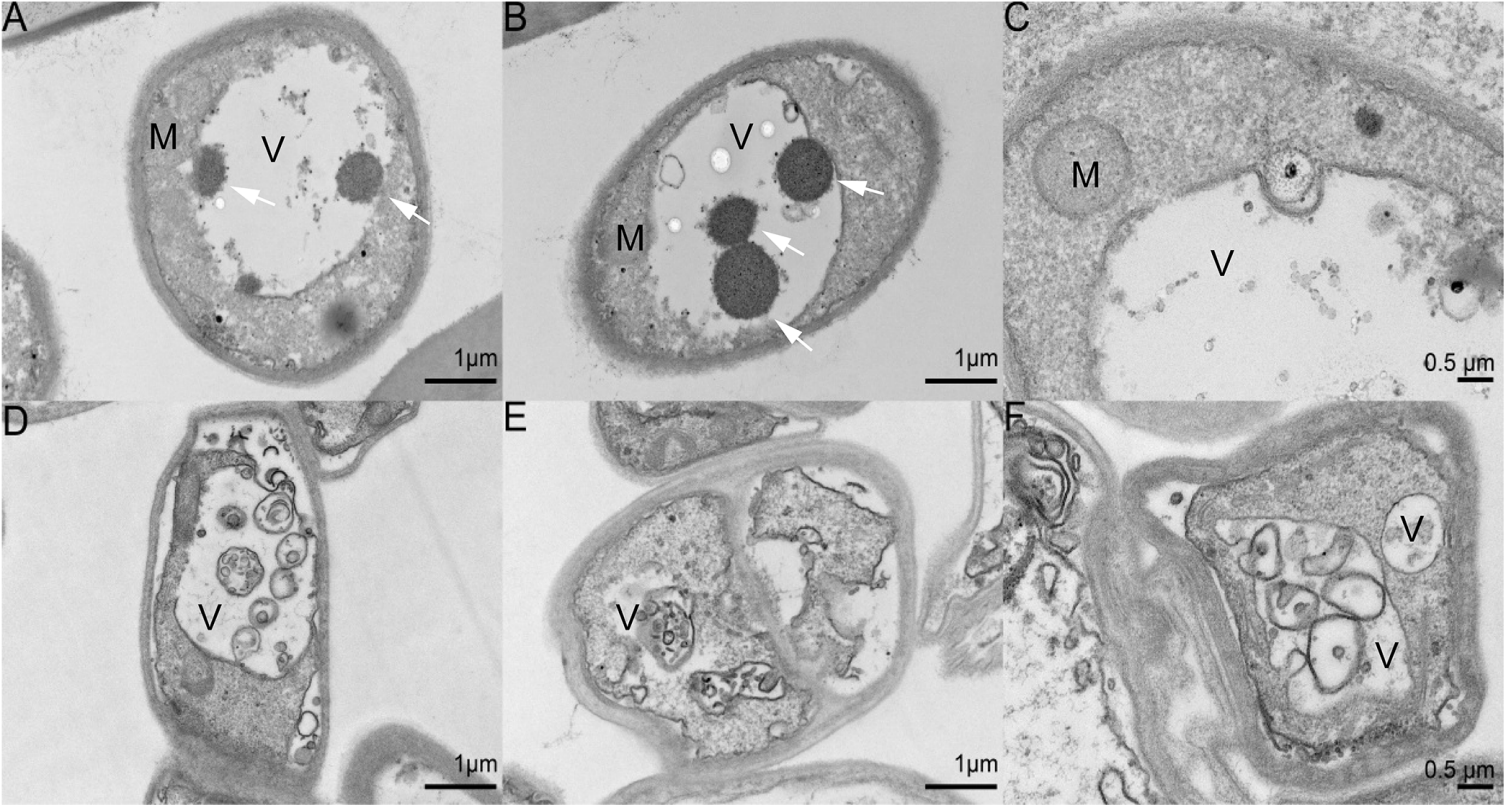
Transmission electron microscopy images of the control (A to C) and TSA-treated (D to F) mycelia. M, mitochondrium; V, vacuole; White arrows, autophagosome.

Protein acetylation is regulated by lysine acetylases and deacetylases (KDACs) (22, 23). To evaluate the role of KDACs in mycelial BF formation and postripening in *L. edodes*, we treated the growing mycelia with TSA, a broad-spectrum KDAC inhibitor, for 30 days. As shown in Fig. 8A, BF formation was delayed in TSA-treated *L. edodes*. Consistent with this, the L value was significantly lower in the TSA-treated mycelia compared to the untreated mycelia (Fig. 8B; Table S11). Western blotting assay was performed to analysis the acetylation change, and did not change significantly during BF formation and postripening of the TSA-treated mycelium (Fig. S6). This result suggest that the protein acetylation inhibitors may have many ways of action, and there are many kinds of deacetylase in the mycelium of *L. edodes*. And the tests were carried out with other different concentrations of TSA (5 μM, 15 μM, 20 μM, 25 μM), and the results also showed that TSA had a significant effect on delaying BF formation and postripening. Furthermore, the inhibition effect of these concentrations is basically the same, which L value has no significant difference (Fig. S7 and S8). Furthermore, the CS and ICDHm activity (Fig. 8C and D; Table S11), along with downregulating *atg8* mRNA expression (Fig. 8E; Table S11). Thus, inhibition of KDACs during mycelial growth also stalled autophagic progression compared to that in the control group. In line with this hypothesis, TEM examination of the TSA-treated and control mycelia revealed significantly larger autophagic vacuoles in the control group compared to the TSA-treated group, which was indicative of vacuolar fusion. Furthermore, the vacuoles in the control group were overall free of cellular content, which indicated that degradation had occurred. We also observed accumulation of autophagosomes in the vacuoles, which are signs of the late stage of autophagy (Fig. 9A to C). In the TSA-treated group, there were numerous vacuoles filled with degraded organelles and other substances, and there were no autophagosome, which is indicative of the early stage of autophagy (Fig. 9D to F). Taken together, blocking KDACs in the growing mycelia inhibited the autophagy flux, which indicates that protein acetylation regulates autophagy and BF formation in *L. edodes*.

## DISCUSSION

### Protein acetylation during mycelial BF formation and postripening in *L. edodes*.

Protein KAC is an important and highly conserved PTM with different biological functions in eukaryotes and prokaryotes. However, relatively little is known regarding the significance of PTM in large edible fungi (24). Our study is the first to explore the lysine acetylome of *L. edodes* mycelia during BF formation and postripening, and evaluate the role of KAC during these processes. A total of 1,261 sites in 741 proteins were differentially lysine-acetylated in the mycelia with BF formation (day 60) compared to that lacking BF (day 30). Most of these proteins had a single KAC site, whereas others like heme peroxidase (15 sites), fatty acid synthase (FAS, 10 sites), and histone H2B (10 sites) were acetylated at multiple lysine positions. Heme peroxidase degrades hydrogen peroxide, oxidizes lignin, and removes pigments, among other functions (25). FAS catalyzes the synthesis of palmitic acid, which is a source of energy and essential for cellular membrane synthesis, protein acylation, signal transduction, and basic cell structural integrity (26). Histone H2B is a chromosomal protein, and H2B acetylation has epigenetic effects on gene expression (27). Thus, mycelial BF formation and postripening in *L. edodes* are accompanied by extensive changes in histone and nonhistone protein KAC.

Light is an important factor influencing mycelial BF formation and postripening in *L. edodes*. The blue light receptors white collar-1 and white collar-2, and the red light receptor phytochromes are significantly upregulated during BF formation (5). Consistent with this, we detected increased acetylation at K130 and K173 in white collar-1, which may be related to its activity. KEGG pathway analysis of the acetylome revealed significant enrichment of the proteasome pathway during BF formation. Previous studies have shown that protein acetylation can regulate proteosome stability and function. For example, acetylation of the α6, β3, β6, and β7 proteasome subunits increases their activity (28). The ubiquitin/proteasome pathway (UPP) degrades nonfunctional or aberrantly folded proteins in eukaryotic cells, and is essential for maintaining cellular homeostasis and survival. In addition, proteosome-dependent degradation is involved in various physiological processes, such as photomorphogenesis, senescence and the stress response. Marrocco et al. (29) found that the photoreceptor PHY is degraded through the UPP pathway during photomorphogenesis in plants. Furthermore, knocking out the proteasome regulatory granule subunit RPN10 significantly delayed the onset of leaf senescence in Arabidopsis thaliana (30). High proteasome activity has been detected in the senescent leaves of wheat and rapeseed (31, 32). We compared the proteasome activity in mycelia before and after BF formation, and found significantly higher activity in the brown mycelia (day 60) compared to that in early white mycelia (day 30). Taken together, the proteasome may play a role in controlling protein quality, cellular homeostasis, and BF formation in the *L. edodes* mycelium.

The TCA cycle plays an important role in carbohydrate metabolism (33). Acetylation of the TCA cycle enzymes is known to modulate their catalytic activities (34). Wang et al. (35) found that the deacetylase Sirt regulates pupal diapause in Helicoverpa armigera by decreasing the acetylation levels of pyruvate kinase, phosphoglucomutase, and phosphoenolpyruvate carboxy kinase, which in turn affects pyruvate synthesis and TCA cycle activity. Deacetylation of malate dehydrogenase (MDH) in *A. thaliana* also reduced TCA activity (36). Exposure of liver cells to high concentrations of glucose increased the acetylation of MDH, which enhanced enzyme activity and accelerated progression through the TCA cycle (37). Furthermore, Hebert et al. (38) also found that acetylation of multiple sites increased MDH activity. In our study as well, the TCA cycle was significantly enriched in the acetylome of mycelia with BF formation, and most of the enzymes in the TCA cycle were differentially acetylated during this process. In particular, the K298 and K280 positions of CS, and the K512 position of IDH were deacetylated in the post-BF (day 60) relative to the pre-BF (day 30) mycelia. CS is the initial rate-limiting enzyme of the TCA cycle, and its activity directly affects the amount of citric acid (39). ICDHm is the second rate-limiting enzyme in the TCA cycle (40). A previous study has shown that acetylation of CS can reduce its total activity (41). In this study, we found that CS and ICDHm activity was significantly lower in the mycelia on day 60 compared to that on day 30. The decreased activity of these enzymes suggests that the TCA cycle is regulated in the late stage of BF formation. Taken together, these findings indicate that the TCA cycle may be a physiological indicator of BF formation.

### Inhibition of KDACs delayed autophagy during mycelial BF formation and postripening in *L. edodes*.

A previous study showed that histone deacetylase inhibitors such as TSA can promote autophagy by increasing global protein acetylation (42). We found that the broad-spectrum KDAC inhibitor TSA delayed mycelial BF formation and stalled autophagic progression in *L. edodes*, indicating that induction of autophagy may shorten the mycelial browning process, which is consistent with previous studies (17). Autophagy is a stress response to various environmental and metabolic signals (43, 44). Acetylation modification is involved in every important processes of autophagy. The acetylation modification of histones and transcription factors can regulate the expression level of autophagy-related genes, and the acetylation/deacetylation modification can regulate the activity of autophagy-related proteins, which can rapidly and accurately regulate autophagy (23). Histone acetylation plays an important role in inducing autophagy in response to long-term nutritional deficiency or stress stimulation. The relationship between autophagic activity and the acetylation of lysine at the 16th position of H4 (H4K16ac) and the 56th position of H3 (H3K56ac) has been studied (45). ATG proteins are important regulatory proteins of autophagy, and many ATG proteins can be acetylated. Studies have shown that the deacetylation of LC3 leads to the nucleation of LC3, which is a necessary condition for initiating autophagy in the process of autophagy formation (46). In yeast, ATG3 can be acetylated by acetylase Esa1, and acetylated ATG3 initiated autophagy by enhancing the interaction between ATG3 and ATG8 (47, 48). In this study, we also found that autophagy-related proteins were also acetylated, and acetylation modification was also closely related to autophagy by deacetylase inhibitors. These results also showed that the acetylation of protein also regulated autophagy of *L. edodes* mycelium. Taken together, these findings confirm the important role of protein acetylation and autophagy on mycelial BF formation and postripening in *L. edodes*. Thus, autophagy may be a physiological feature of mycelial browning that can be used to determine the status of BF formation. We will subsequently focus on whether the period of mycelial browning, and eventually the cultivation cycle of *L. edodes*, can be shortened by inducing autophagy.

To summarize, we analyzed the lysine acetylome during mycelial BF formation and postripening of *L. edodes*, and determined the influence of protein acetylation and autophagy on these processes. Our findings provide new insights into the mechanisms underlying mycelial BF formation and postripening, which may have far-reaching significance for the cultivation and production of *L. edodes*.

## MATERIALS AND METHODS

### Fungal cultivation.

The *L. edodes* KS11 strain and cultivation bags were purchased from Shanghai Chengying Agriculture Development Co. Ltd. The fungus was cultured in 1,000 g cultivation medium (32% dry sawdust, 8% dry corn bran, and 60% water) at 22°C in the dark for 30 days until the substrate was fully colonized by white mycelia. The bags were then subjected to a 12 h light/dark cycle (white light, 300 lx) for 30 days to induce BF formation on the mycelial surface. Samples of white films (day 30) and BFs (days 45, 60, 75) were collected from the surface of mycelia, snap-frozen in liquid nitrogen, and stored at −80°C.

### Protein extraction and Western blotting.

The frozen mycelial film samples were ground to powder form in liquid nitrogen and then transferred to a 5 mL centrifuge tube. The cell powders were suspended in four volumes lysis buffer (8 M urea, 1% Triton-100, 10 mM dithiothreitol, and 1% protease inhibitor cocktail), and sonicated three times on ice using a high-intensity ultrasonic processor (Scientz). The remaining debris was removed by centrifuging at 20,000 × *g* for 10 min at 4°C. Finally, the protein was precipitated with cold 20% TCA for 2 h at −20°C, and centrifuged at 12,000 × *g* for 10 min at 4°C. The supernatant was discarded, and the precipitate was washed three times with cold acetone. The protein was redissolved in 8 M urea, and its concentration was determined with a BCA kit according to the manufacturer’s instructions.

### Enrichment of lysine-acetylated peptides.

The protein solution was reduced with 5 mM dithiothreitol for 30 min at 56°C and alkylated with 11 mM iodoacetamide for 15 min at room temperature in the dark. After diluting with 100 mM TEAB and urea to less than 2 M, the samples were first digested overnight with trypsin at the 1:50 trypsin/protein mass ratio, and then for 4 h with 1:100 trypsin. The tryptic peptides were dissolved in NETN buffer (100 mM NaCl, 1 mM EDTA, 50 mM Tris-HCl, 0.5% NP-40; pH 8) and incubated overnight with pre-washed antibody beads (Cat. No. 001, PTM Bio) at 4°C with gentle shaking. The beads were washed four times with NETN buffer and twice with water. The bound peptides were eluted from the beads with 0.1% trifluoroacetic acid. All eluted fractions were combined and vacuum-dried, and then desalted with C18 ZipTips (Millipore) for LC-MS/MS analysis according to the manufacturer’s instructions.

### LC-MS/MS analysis.

The tryptic peptides were dissolved in 0.1% formic acid (solvent A) and directly loaded onto a self-made reversed-phase analytical column (15 cm length, 75 μm i.d.). The gradient consisted of 6% to 23% solvent B (0.1% formic acid in 98% acetonitrile) in 26 min, 23% to 35% in 8 min, and to 80% in 3 min. For the last 3 min, 80% solvent B was used at the constant flow rate of 400 nL/min on an EASY-nLC 1000 UPLC system.

The peptides were subjected to an NSI source followed by MS/MS in a Q Exactive Plus system (Thermo Fisher Scientific) coupled online to the UPLC. The electrospray voltage was 2 kV. The *m/z* scan range was 350 to 1,800 for the full scan, and intact peptides were detected in the Orbitrap at a resolution of 70,000. Peptides were then selected for MS/MS using an NCE setting of 28, and the fragments were detected in the Orbitrap at a resolution of 17,500. A data-dependent procedure was used that alternated between one MS scan followed by 20 MS/MS scans with 15-s dynamic exclusion. The automatic gain control (AGC) was set at 5E4 and the fixed first mass was set as 100 *m/z*.

### Database search.

The resulting MS/MS data were processed using the MaxQuant search engine (v.1.5.2.8, http://www.maxquant.org/). Tandem mass spectra were searched against the *L.* edodes UniProt database (Lentinula_edodes_5353_PR_20190801, 12,046 sequences) concatenated with the reverse decoy database. Trypsin/P was specified as a cleavage enzyme, allowing up to two missing cleavages. The mass tolerance for precursor ions was set as 20 ppm in the first search and 5 ppm in the main search, and the mass tolerance for fragment ions was set as 0.02 Da. Carbamidomethyl on Cys was specified as a fixed modification, and acetylation modification and oxidation on Met were specified as variable modifications. False discovery rate (FDR) thresholds for protein, peptide, and modification sites were specified at 0.01. The minimum peptide length was set at 7 to identify acetylated lysine sites with a localization probability less than 0.75 or reverse, and contaminating protein sequences were removed.

### Bioinformatics analysis.

**(i) Protein annotation and functional classification.** The GO annotation proteome was derived from the UniProt-GOA database (http://www.ebi.ac.uk/GOA/). The protein IDs were converted to UniProt IDs and then mapped to GO IDs. In case the identified proteins were not annotated by the UniProt-GOA database, InterProScan software (v.5.14-53.0, http://www.ebi.ac.uk/interpro/) was used for annotation based on the protein sequence alignment method (49). The proteins were classified based on biological processes, cellular components, and molecular functions.

The protein domains were annotated by InterProScan based on the protein sequence alignment method using the InterPro database (http://www.ebi.ac.uk/interpro/), which integrates the information of protein families, domains, and functional sites via web-based interfaces (50, 51).

The protein pathways were annotated using the KAAS online tool (v.2.0, http://www.genome.jp/kaas-bin/kaas_main) of the Kyoto Encyclopedia of Genes and Genomes (KEGG) database (52), and the results were mapped to the database using the KEGG mapper (V2.5, http://www.kegg.jp/kegg/mapper.html). Finally, subcellular localization of the proteins was predicted using WoLF PSORT (v.0.2, http://www.genscript.com/psort/wolf_psort.html), an updated version of PSORT/PSORT II for the prediction of eukaryotic sequences (53).

**(ii) Motif analysis.** Soft MoMo (motif-x algorithm) (V5.0.2, http://meme-suite.org/tools/momo) was used to analyze the positions of modify-21-mers (10 amino acids upstream and downstream of the acetylated site) in all protein sequences. All the protein sequences in the database were used as background parameters. The minimum number of occurrences was set to 20. The original motif-x option was checked, and other parameters were set to default (54).

**(iii) Functional enrichment analysis.** The acetylated proteins were functionally annotated by GO, KEGG pathway and domain analyses using the functional enrichment tool of the Perl module (v.1.31, https://metacpan.org/pod/Text::NSP::Measures::2D::Fisher). The enrichment of the differentially modified proteins was checked against all identified proteins using the two-tailed Fisher's exact test. Correction for multiple hypotheses testing was performed using standard FDR control methods (55), and *P* < 0.05 was considered significant (52).

### Proteasome activity assay.

Mycelia protein was extracted and proteasome activity was analyzed using the method of Gong et al. with slight modifications (56). Briefly, the frozen samples were ground in liquid nitrogen and homogenized in lysis buffer (500 μL for 300 mg powder, 50 mM HEPES pH 7.6, 15 0 mM NaCl, 1 mM EDTA, 1% Triton X-100, and 15% glycerol). The homogenates were lysed by sonicating at 4°C for 20 min and then centrifuged at 12,000 rpm for 10 min at 4°C. To assay the proteasome activity, 10 μL supernatant was mixed with the reaction buffer (final concentration: 25 mM HEPES pH 7.5, 0.5 mM EDTA, 0.05% NP-40, and 0.001% SDS wt/vol), and the fluorogenic peptide substrate Suc-LLVY-AMC (Merck Millipore; reconstituted with dimethyl sulfoxide) was added at the final concentration of 1 mM. The reaction (total volume 100 μL) was conducted in a 96-well fluorometer plate for 2 h at 37°C. The fluorescence intensity was calculated at 360 nm excitation and 460 nm emission. For the inhibition assay, 40 μM MG123 was pre-incubated with proteasome extracts for 15 min at room temperature, and then the Suc-LLVY-AMC substrate was added.

### CS and ICDHm activity assay.

CS and ICDHm enzyme activity assays were performed using the specific assay kits, (#CS-1-Y, #ICDHM-1-Y, Omin, Suzhou, China) according to the manufacturer's instructions. The contents of citrate, isocitrate, alpha-ketoglutarate, and succinate were determined by Shanghai Applied Protein Technology Co. Ltd.

### TCA intermediates quantification.

The samples were frozen in liquid nitrogen and ground with a pestle and mortar. Taking 100 mg sample, mixed with 1 mL of cold methanol/acetonitrile/H_2_O (2:2:1, vol/vol/vol), the homogenate was sonicated at low temperature (30 min/once, twice). The mixture was centrifuged for 20 min (14,000 g, 4°C). The supernatant was dried in a vacuum centrifuge. HPLC analysis were performed using an UHPLC (1290 Infinity LC, Agilent Technologies) coupled to a QTRAP (AB Sciex 5500). For HILIC separation, samples were analyzed using an ACQUITY UPLC BEH Amide column (2.1*100 mm, 1.7 μm, Waters MS Technologies, Manchester, UK). The mobile phase contained A = 15 mM CH3COONH4 in water and B = acetonitrile. The samples were in the automatic sampler at 4°C, and the column temperatures were kept constant at 45°C, the gradients were at a flow rate of 300 μL/min, and a 2 μL aliquot of each sample was injected. In ESI negative modes, the conditions were set as follows: source temperature 450°C, ion Source Gas1(Gas1): 45 lb/in^2^, ion Source Gas2 (Gas2): 45 lb/in^2^, curtain gas (CUR): 30 lb/in^2^, ionSapary Voltage Floating (ISVF) -4500V. Data acquisition and processing were accomplished using Multiquant software (57).

### Trichostatin A treatment.

Cultivation bags of *L. edode*s were incubated at 22°C in the dark for 30 days till the mycelia grew all over. The bags were divided into three groups, and respectively injected with 5, 10, 15, 20, 25 μM TSA, 0.5%, 1%, 1.5%, 2%, 2.5% DMSO, or untreated as blank control. After culturing for 30 days under alternating 12 h of light (white light, 300 lx) and 12 h of darkness, the samples were collected for further analysis.

### Transmission electron microscopy.

The mycelial samples were prepared as described by the method of He et al., and cut into 1-mm blocks by an ultramicrotome (Leica EM UC7, Wetzlar, Germany) for a JEM-2100 transmission electron microscopy (JEOL, Tokyo, Japan) observation (58).

### Quantitative RT-PCR.

Total mycelial RNA was extracted from TSA and CK samples using a TRIzol kit (Invitrogen, USA) and reverse-transcribed to cDNA using a TaKaRa reverse transcription kit. The primers for *atg8* and *β-actin* (internal control) were designed using the Primer Premier 6 software. Fluorescent qRT-PCR was performed on the Model 7500 cycler (Applied Biosystems, Foster City, CA, USA). Three biological replicates were set up for each sample, and the relative gene expression level was calculated using the 2^-ΔΔCT^ method and converted to a log_2_FC value.

### Data availability.

The mass spectrometry proteomics data have been deposited to the ProteomeXchange Consortium (http://proteomecentral.proteomexchange.org) via the iProX partner repository with the dataset identifier PXD042811.
