# Supplementary material for: Potential Role of Lysine Acetylation and Autophagy in Brown Film Formation and Postripening of Lentinula edodes Mycelium
Source: Microbiol Spectr. 2023 Jun 22;11(4):e02823-22. doi: 10.1128/spectrum.02823-22 (PMC10434168; doi:10.1128/spectrum.02823-22)
Supplement: Supplemental file 3 — Figures S1-S8; Tables S3 and S10. Download spectrum.02823-22-s0001.pdf, PDF file, 1.1 MB [file spectrum.02823-22-s0001.pdf]

Potential role of lysine acetylation and autophagy in brown film formation and post-ripening of *Lentinula edodes* mycelium

Ting Chu[1, 2], Junjun Shang[1], Huahua Jian[3], Chunyan Song[1], Ruiheng Yang[1], Dapeng Bao[1], Qi Tan[1], Lihua Tang[1*]

[1]National Engineering Research Centre of Edible Fungi, Key Laboratory of Edible Fungi Resources and Utilization (South), Ministry of Agriculture, Institute of Edible Fungi, Shanghai Academy of Agricultural Sciences, Shanghai 201403, China
[2]School of Food Sciences and Technology, Shanghai Ocean University, Shanghai 201306, China
[3]State Key Laboratory of Microbial Metabolism, School of Life Sciences and Biotechnology, Shanghai Jiao Tong University, Shanghai 200240, China

***Correspondence:**
Email: lhtang2012@163.com (L.H.);

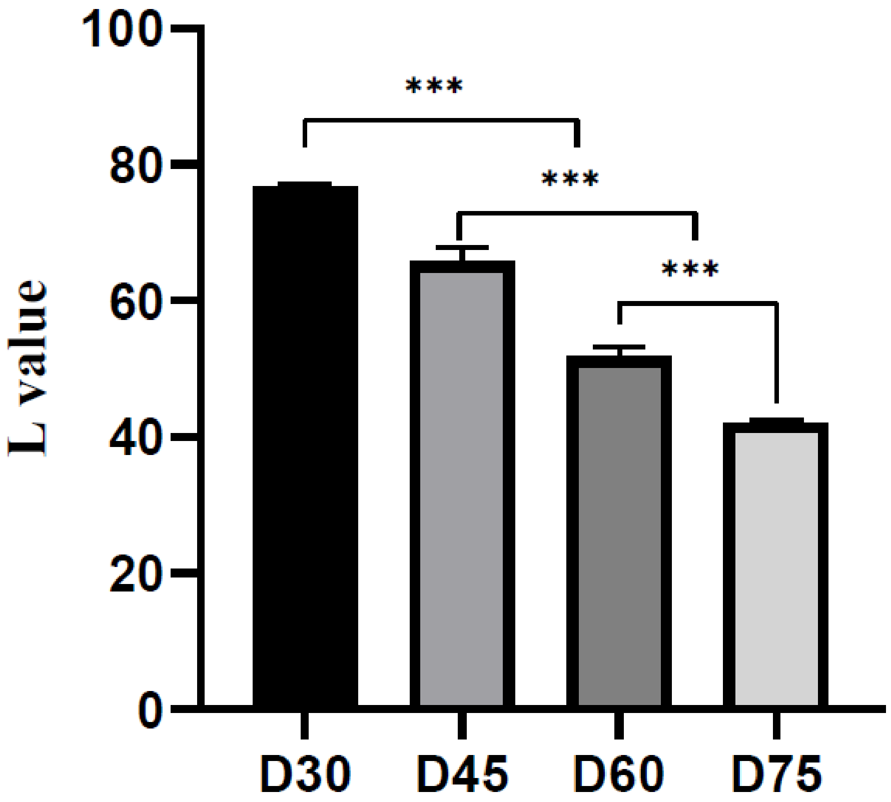

Supplementary Figure S1. The anova test of these L value among the four time points

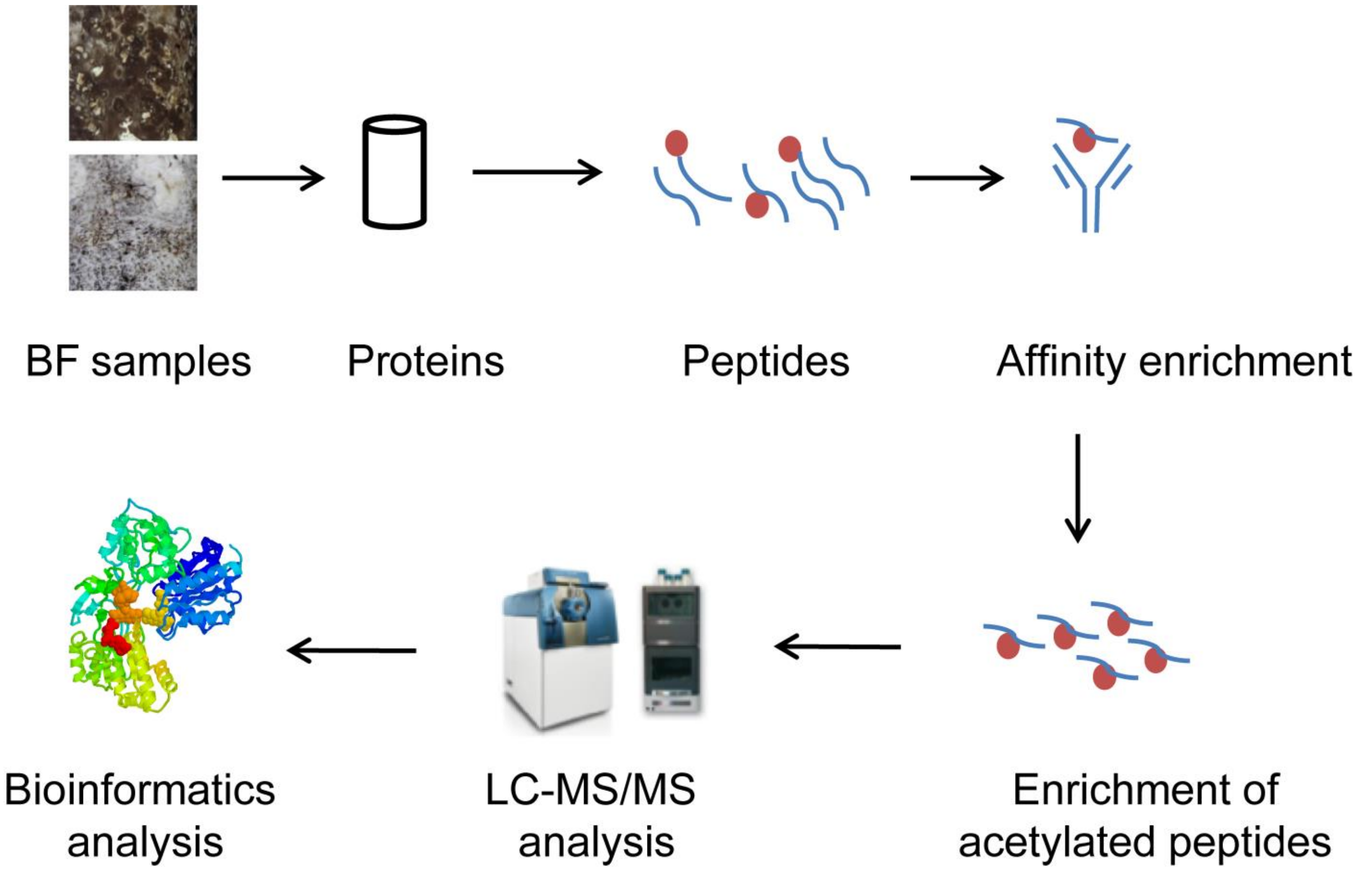

Supplementary Figure S2. The workflow for the acetylome analysis of myclium in *L.edodes*. LC-MS/MS: liquid chromatography-tandem mass spectrometry.

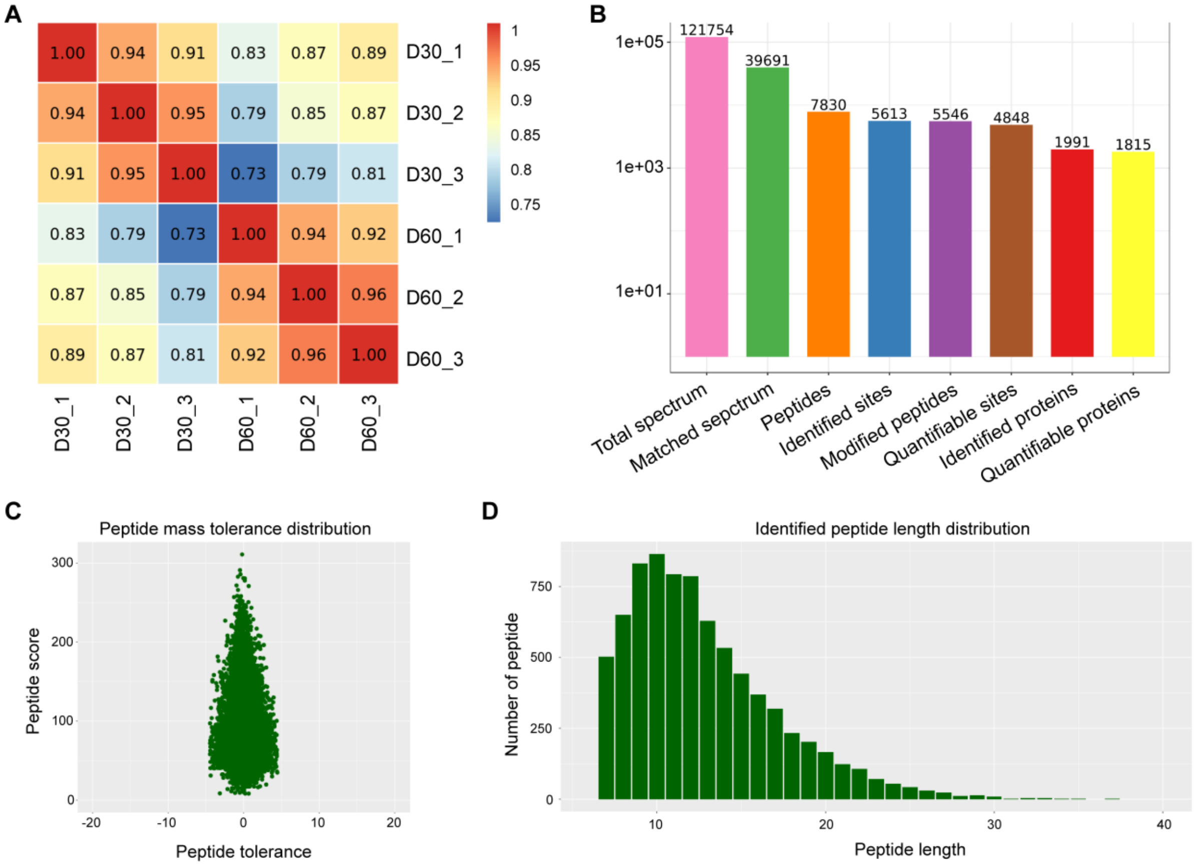

Supplementary Figure S3. Overview of acetylated proteomes. (A) Pearson's correlation of the acetylation proteomes from two sample groups (three biological replicates for each group). (B) Quantification overview of acetylated sites and proteins from the mass spectrum. (C) Mass error distribution of all identified acetylated peptides. (D) Length distribution of all identified acetylated peptides.

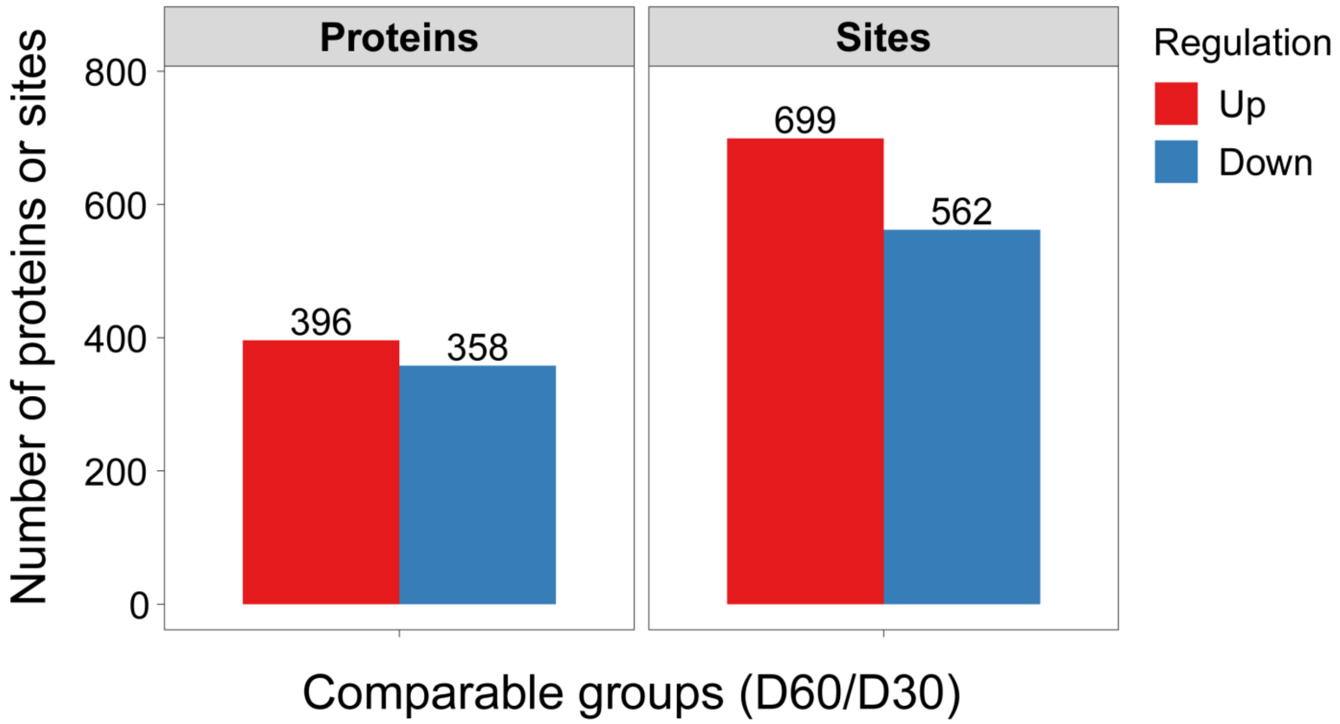

Supplementary Figure S4. Statistical analysis of differential acetylated sites and proteins in day 30 (D30) and day 60 (D60) comparison.

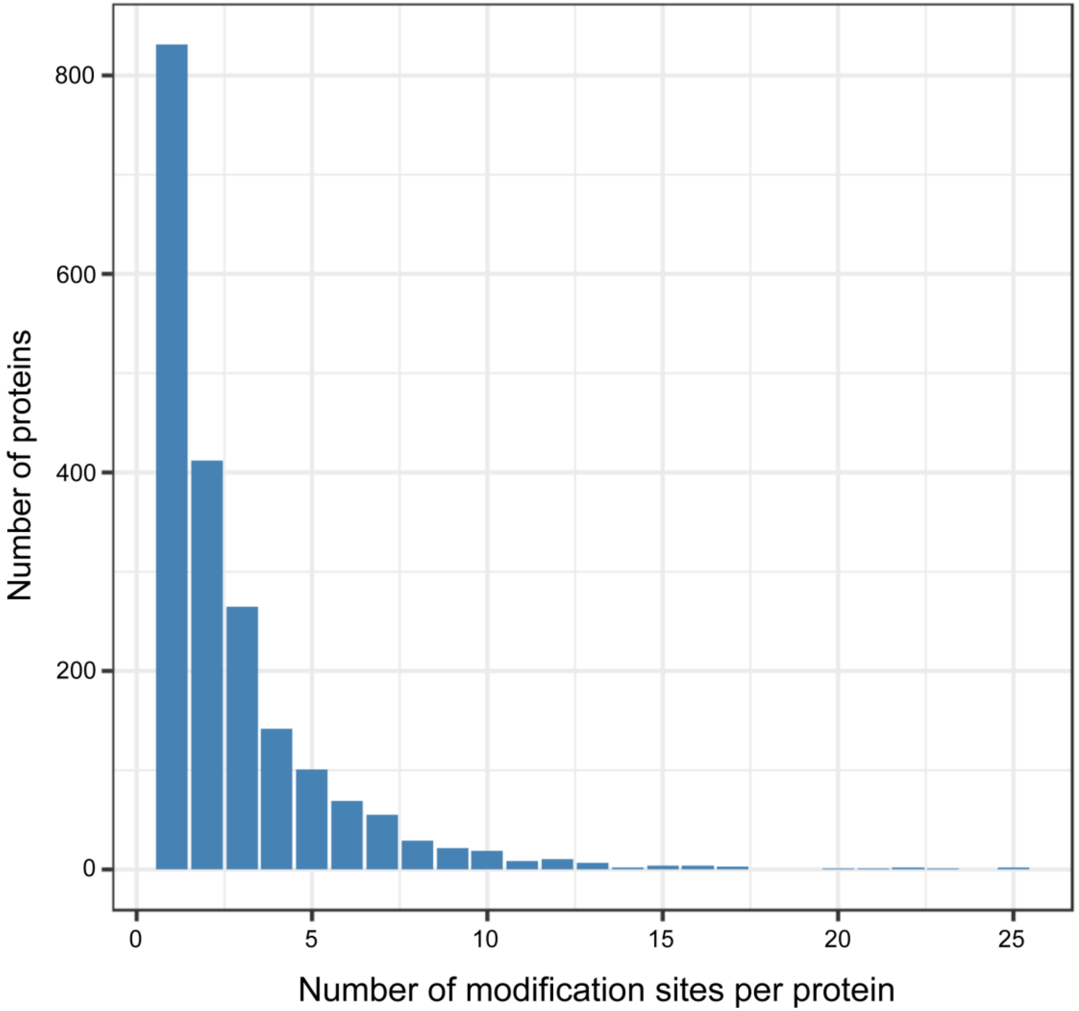

Supplementary Figure S5. The number of KAC sites in these proteins

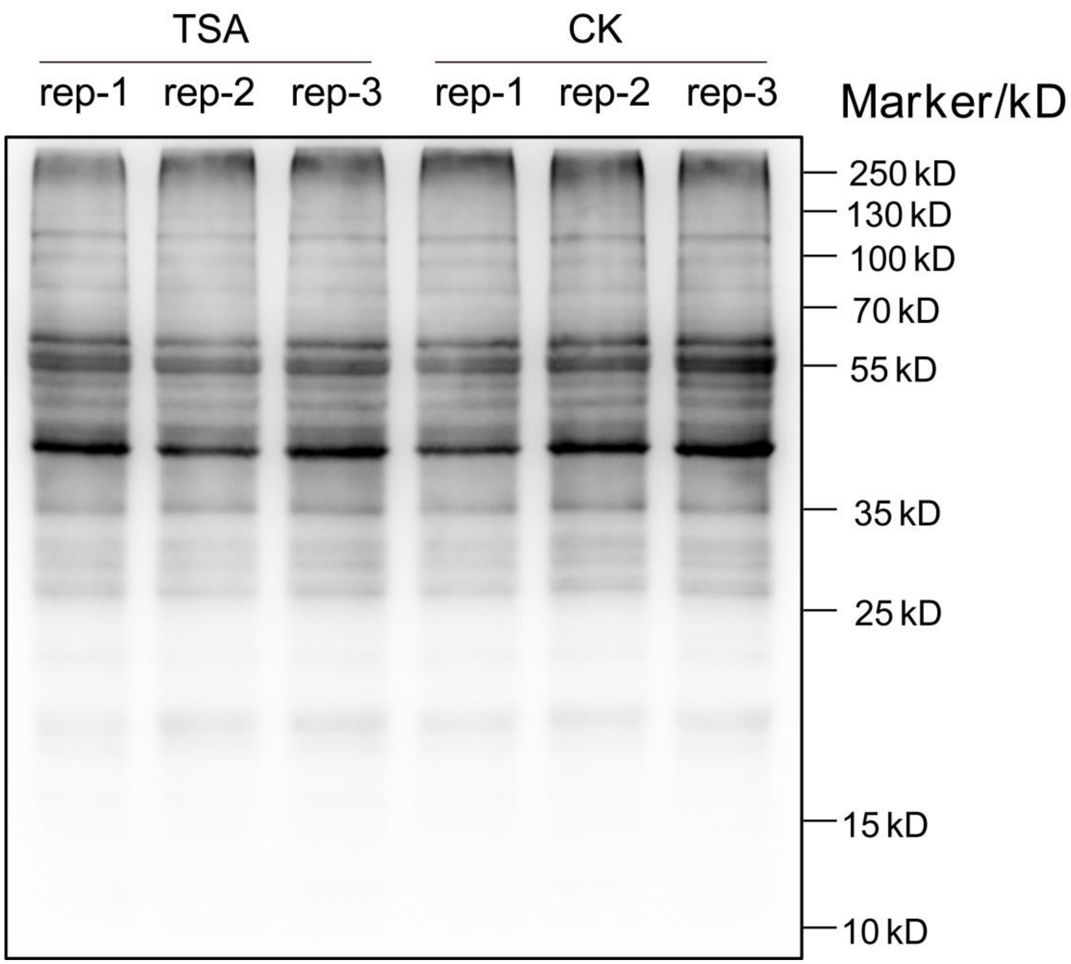

Supplementary Figure S6. Immunoblot showing levels of acetylated proteins between the TSA-treated mycelia compared to the untreated mycelia

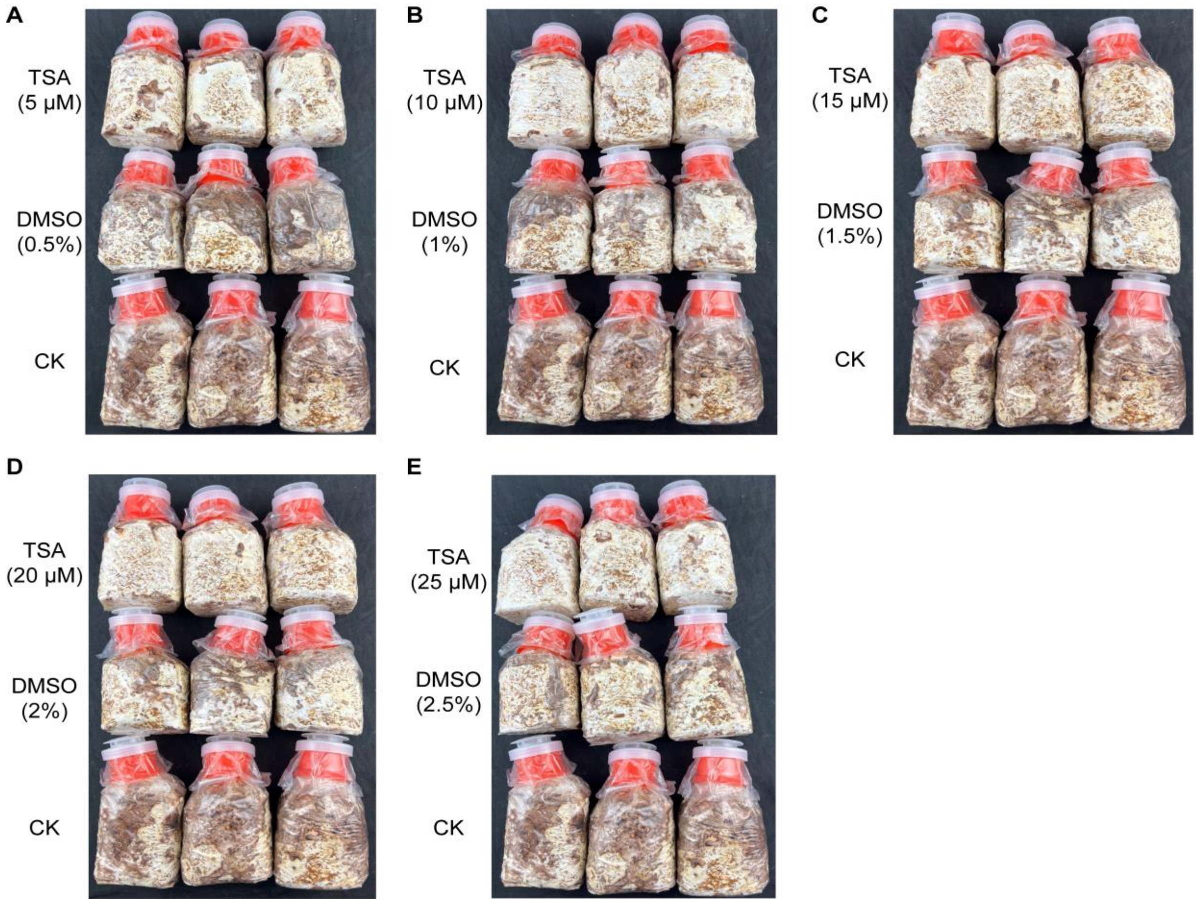

Supplementary Figure S7. Representative images showing BF formation in TSA-treated (5µM, 10µM, 15µM, 20µM, 25µM), control (CK) and DMSO-treated groups.

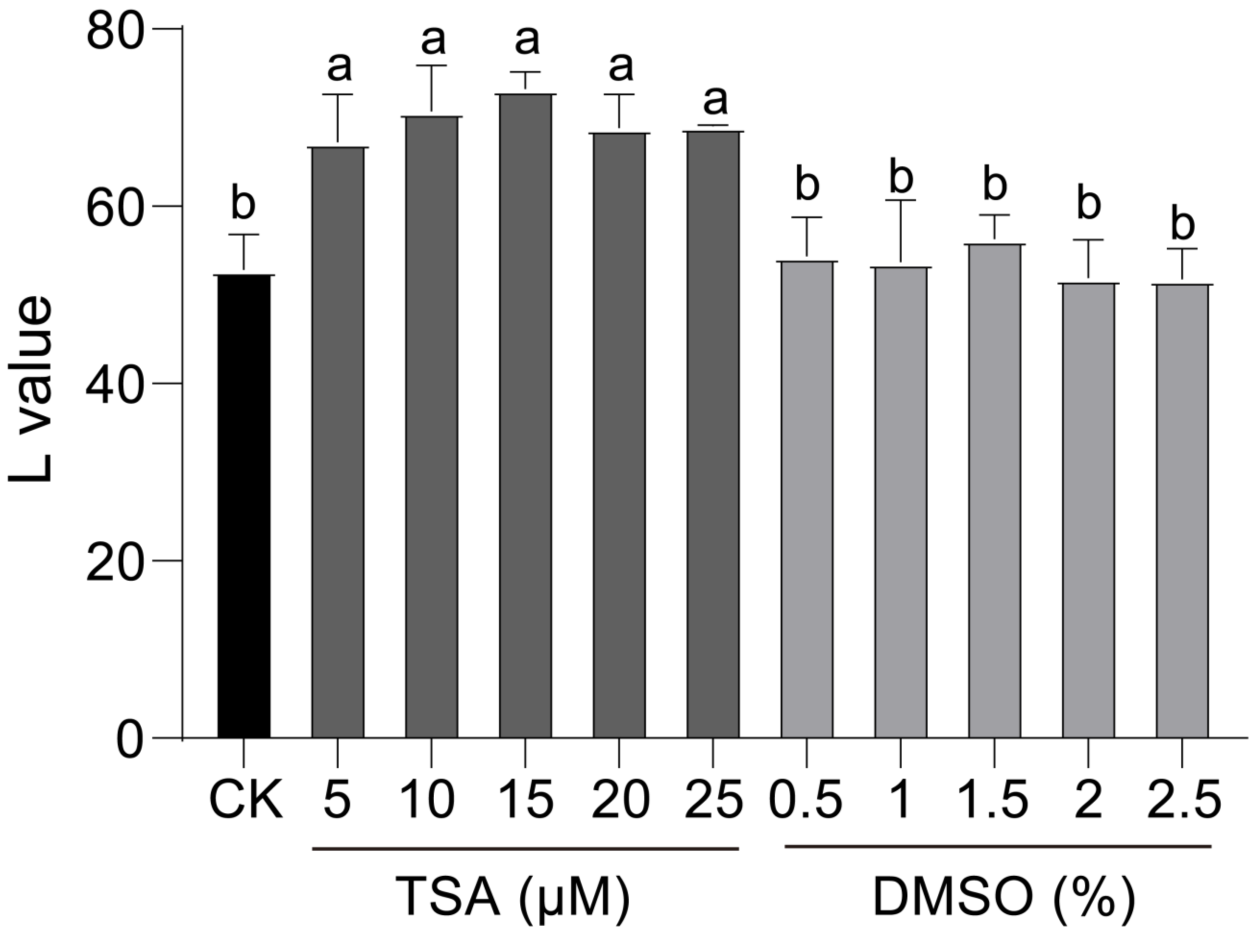

Supplementary Figure S8. The L value of different concentrations of TSA-treated mycelium (5μM, 10μM, 15μM, 20μM, 25μM)

Supplementary Table S3. The heatmap of the amino acids around the acetylation sites

Amino	-10	-9	-8	-7	-6	-5	-4	-3	-2	-1	K	1	2	3	4	5	6	7	8	9	10
A	1.5	0.07	2.98	1.28	1.49	0.9	-0.02	1.09	3.7	3.04	0	-5	0.48	1.21	0.1	0.3	1.37	0.44	-0.17	1.61	0.32
C	-1.35	-1.4	-5	-0.67	-0.48	-0.25	-0.27	0.05	-0.22	-1.64	0	-5	-0.57	-1.05	0.08	-0.13	-1.91	-0.8	-0.22	-1.12	-2.24
D	-0.04	-1.39	-0.96	0	-0.89	-0.53	-0.05	-2.02	-1.12	-0.16	0	-5	3.5	-0.7	-0.32	-0.79	-0.87	-0.84	-0.59	-0.13	-0.42
E	-0.74	-2.03	-0.46	-0.26	-1.54	-0.7	-0.42	0.05	-0.47	-4.78	0	-5	2.33	-2.67	0.07	-1.83	-1.08	-2.93	0	-1.02	-0.53
F	0.33	1.83	0.4	0.95	1.18	-0.29	0.18	5	3.69	5	0	5	5	2.04	0.52	-0.03	-0.16	0.18	0.35	-0.08	0.11
G	0.61	1.87	0.54	0.68	3.02	1.62	1.01	0.4	3.53	2.81	0	-5	1.11	1.03	0.1	0.58	4.05	1.37	1.74	5	2.18
H	0.05	0.36	0.79	-0.17	0.19	5	5	5	5	3.8	0	5	5	5	1.75	0.62	1.26	0.94	-0.26	-0.54	1.15
I	0.91	1.49	1.09	1.27	3.06	0.06	1.88	1.69	4.1	5	0	-5	0.4	4.24	0.72	0.12	0.56	1.3	0.68	0.3	1.22
K	2.83	5	5	5	3.2	5	-0.17	-2.23	-3.99	-5	0	-2.01	-5	0.12	0.52	5	4.77	5	5	5	4.47
L	-0.18	-0.24	-2.19	-1.04	-0.03	-0.44	0.4	2.83	3	1.91	0	-5	-0.02	-0.01	0.53	-1.07	0.23	0.68	-0.42	-1.48	0.13
M	-0.18	-1.13	-0.98	0.09	0.27	-2.46	-0.3	-2.93	-1.19	-4.77	0	-5	-2.19	-0.9	-2.05	-1.74	-0.31	-0.66	-0.79	-0.02	-0.12
N	-1.34	-1.28	-2.67	-1.29	-1.27	-0.53	-0.26	-1.95	-0.45	1.64	0	5	-0.14	-1.31	-0.13	0.11	0.3	-0.69	0.45	-0.52	-0.03
P	-0.6	-0.53	-0.75	-1.35	-2.16	-2.39	-0.77	-0.94	-4.54	0.09	0	-5	-4.88	-1.27	-0.74	-2.48	-1.38	-2.58	-2.52	-3.31	-1.61
Q	-1.66	-1.29	-1.48	-0.63	-0.41	-0.33	0.14	-1.99	-1.39	-2.93	0	-5	-1.65	0	-0.25	-0.04	-0.7	0.43	-0.61	-0.33	-1.26
R	-0.42	0.44	3.79	1.01	1.45	0.54	0.2	-2.68	-2.56	-5	0	0.93	0.45	0.68	0.39	4.71	0.96	0.59	1.67	1.21	0.99
S	-3.12	-3.27	-3.96	-5	-5	-3.54	-5	-4.92	-5	-0.27	0	5	-5	-5	-5	-3.14	-5	-5	-5	-5	-3.51
T	0.71	-0.03	-0.84	-1.31	-1.09	0.08	0.1	0.2	-0.98	5	0	5	-0.41	-1	-0.13	-0.22	-0.09	-0.05	-0.46	-0.42	-1.16
V	1.18	2	2.93	0.26	0.5	0.96	1.04	0.51	1.69	5	0	-0.77	-0.22	2.25	3.17	0.72	0.22	1.33	2.56	1.92	0.15
W	-0.14	0	-1.86	-0.36	-0.34	-3.61	-0.87	-0.43	0.53	-4.74	0	5	0.9	-0.11	0	-0.41	-1.08	-0.09	-2.64	0.28	-1.86
Y	1.4	-0.02	0.08	1.55	0.77	1.52	0.78	5	2.68	5	0	5	3.21	4.47	1.12	2.44	1.42	1.58	2.56	1.19	4.82

Supplementary Table S10 The results of Metabolite content in TCA cycle

Metabolite Name	KEGG ID	Transitions	Retention Time(min)	QC RSD	day30-1	day30-2	day30-3	day30-4	day30-5	day30-6	day60-1	day60-2	day60-3	day60-4	day60-5	day60-6	QC-1	QC-2	QC-3
alpha-keto glutarate	C00026	145.2 / 101.1	3.6943275	0.1022373	19.279055	11.183055	9.2835564	9.1892909	9.5422395	11.176596	25.901001	38.093034	86.354754	30.67058	69.655216	74.978764	39.784257	48.609456	42.909991
Succinate	C00042	117.1 / 73.0	4.5524673	0.1634193	40.418382	29.273138	28.548719	32.726708	17.388444	16.515866	65.737466	243.19936	401.29628	164.24364	382.52699	511.84429	157.688	195.28343	219.59338
Isocitrate	C00311	191.0 / 73.0	7.3249999	0.0760575	3.0894165	2.557458	2.1307506	3.3254088	2.0401206	1.8425151	4.2493513	5.9694662	11.243362	4.0962058	15.724607	5.7299346	4.6260678	5.3593687	4.8479035
Citrate	C00158	191.1 / 87.1	7.4894037	0.0874307	43.407591	27.98365	17.093326	35.433867	13.486643	15.722818	72.173199	199.19317	341.62976	156.10572	404.7665	191.19271	121.75698	142.6992	124.68374
